# An example of land-use relaxation and its effect on soil stabilization in the catchment of Lake Lavijärvi, Russian Karelia: insights from multi-proxy sediment analysis

**DOI:** 10.1007/s10933-026-00394-2

**Published:** 2026-06-05

**Authors:** Mischa Haas, Fatemeh Ajallooeian, Natalya A. Belkina, Dmitry A. Subetto, Negar Haghipour, Caroline Welte, Carsten J. Schubert, Timothy Ian Eglinton, Nathalie Dubois

**Affiliations:** 1 Department of Surface Waters Research and Management, Eawag, Überlandstrasse 133, 8600 Dübendorf, Switzerland; 2 Department of Earth and Planetary Sciences, ETH Zürich, Sonneggstrasse 5, 8006 Zurich, Switzerland; 3 Herzen State Pedagogical University of Russia, Emb. River Moika 48, Saint Petersburg, Russia 191186; 4 Northern Water Problems Institute, Russian Academy of Sciences, A. Nevsky Str. 50, Petrozavodsk, Russia 185003; 5 Laboratory of Ion Beam Physics, ETH Zürich, Otto-Stern-Weg 5, 8093 Zurich, Switzerland; 6 Department of Surface Waters Research and Management, Eawag, Seestrasse 79, 6047 Kastanienbaum, Switzerland; 7 Department of Environmental System Sciences, ETH Zürich, Universitätstrasse 16, 8092 Zurich, Switzerland; 8 Institute of Biogeochemistry and Pollutant Dynamics, Federal Institute of Technology Zurich (ETHZürich), Zurich, 8092 Switzerland

**Keywords:** Land abandonment, Radiocarbon anomalies, Sediment geochemistry, Leaf waxes, Compound-specific radiocarbon dating

## Abstract

Accelerated soil erosion due to land use is a far-reaching problem for today’s society. Soil degradation leads to outwash and redistribution of soil organic matter, which not only impacts soils but also adjacent ecosystems by downstream eutrophication of freshwater bodies. Despite the severity of the problem and the ever-growing footprint of agriculture, little is known about the long-term effects of past soil degradation periods on catchment soils and lake systems. This study addresses this question by analyzing a sediment sequence retrieved from Lake Lavijärvi, located near the city of Sortavala, north of Lake Ladoga in the Republic of Karelia, Russia, which has witnessed a period of intensive farming at the beginning of the twentieth century, followed by abandonment of the land post-World War II. We applied a multi-proxy approach using geophysical properties, the geochemical and isotopic composition of the bulk sediment, as well as the distribution of sedimentary leaf waxes and their relative ages (compound-specific radiocarbon dating). With the beginning of industrialization (ca. 1900 CE), we observed severe soil erosion, evident as maxima in magnetic susceptibility and Ti concentrations, as well as high sediment mass accumulation rates. Intensive farming led to a reduction of soil-carbon-residence times (mean transfer time, MTT), indicated by the washout and deposition of pre-aged soil organic carbon. During the same time, the lake became eutrophic and varves were deposited, due to a surplus of soil-derived nutrients. Decreasing MTT after World War II reflected depopulation and land abandonment that resulted in stabilization of catchment soils. Even though soil erosion declined and the trophic status of Lake Lavijärvi returned to more oligotrophic conditions during the post-war period, the lake still suffers from anoxia today. We show that land-use legacies exert a major influence on freshwater ecosystems decades after land-use relaxation.

**Supplementary Information:**

The online version contains supplementary material available at 10.1007/s10933-026-00394-2.

## Introduction

Soil degradation caused by land use is a far-reaching problem for today’s society (Lal 2005). Accelerated soil erosion leads to outwash and redistribution of soil organic matter (OM), which impacts not only the soil ecosystem directly but also adjacent ecosystems indirectly, such as downstream eutrophication of water systems (Rekolainen et al. 2006).

In the last decades, the preservation of the Earth’s finite soil resources and sustainable land use became an important topic not only from an environmental but also from an economical aspect, since the soil-production capacity is highly dependent on soil-ecosystem functions (Adhikari and Hartemink 2016). Even though there is clear awareness of accelerated soil erosion and its consequences for the soil ecosystem, our knowledge about its long-term influence on the soil-carbon dynamics is still limited (Foster et al. 2003; Haas et al. 2019). Due to the loss of material it is challenging to measure erosion directly on the ground, e.g. by radionuclide dating (Mabit et al. 2014), therefore alternative approaches have to be considered. Lakes are natural repositories for erosional products from catchment soils. Their continuous sediment archives integrate a catchment-wide signal and often hold information about past environmental changes or, specifically, the evolution of soil erosion in the catchment (Huang and O’Connell 2000). In this framework, past periods of land use recorded in lake sediment offer valuable case studies to gain important insights into the fate of mobilized soil material or the magnitude and rate of changes in soil stability over the long term (Haas et al. 2019).

Previous paleolimnological studies were able to detect inputs of catchment soils during land-use periods using a variety of geophysical, geochemical or biological methods. The routine use of magnetic susceptibility (MS) (Huang and O’Connell 2000) or the relative abundance of soil specific elements in the sediments determined by X-ray fluorescence scanning (XRF) (Lavrieux et al. 2017) are among the most frequently used methods to detect soil erosion.

In addition to the mineral composition, the geochemical composition of the sedimentary OM can show large differences compared to intervals without enhanced soil input (Meyers 1994; Enters et al. 2006). Sensitive indicators to detect changes in the composition or the source of the sedimentary OM are the total organic carbon content (TOC), the total nitrogen content (TN) or the atomic TOC/TN ratio (C/N) (Enters et al. 2006). In addition, the isotopic composition of the OM, e.g. δ^13^C and δ^15^N, can also vary significantly and may provide source information.

Finally, by analyzing the leaf wax molecular distributions in sediments it is possible to estimate relative amounts of different lipid sources, such as from aquatic, or terrestrial but also petrogenic “fossil” inputs, e.g. from bedrock erosion (Haas et al. 2020). Leaf wax lipids, such as *n*-alkanes or *n*-carboxylic acids, are predominantly produced by higher terrestrial plants and are, due to their persistent molecular structure, well preserved in the sediment record and hold specific paleoenvironmental information (Eglinton and Hamilton 1967; Lavrieux et al. 2017).

Leaf waxes tend to be stabilized through association with mineral surfaces, and hence they tend to reflect export of the mineral-associated fraction of soils (Eglinton et al. 2021).

In more recent studies, radiocarbon dating of different sedimentary organic fractions, such as macrofossils, TOC, but also soil-specific compounds like leaf waxes, has proven to be an effective tool to track soil organic carbon (SOC) and furthermore evaluate transfer times between different OC pools (Douglas et al. 2014, 2018; Gierga et al. 2016). Soil erosion entails mobilization of pre-aged SOC, of which a fraction, particularly that which is mineral associated, is eventually deposited in lake sediments. This circumstance often leads to radiocarbon anomalies in sediment sequences: a bias towards older ages when radiocarbon dated sediments contain soil erosional products (Edwards and Whittington 2001; Haas et al. 2019). Although not much research has been conducted on the topic, the effect may be attributed to a difference in the retention (or transit) times of organic fractions that are deposited in a sedimentary repository. The effect is even more pronounced when comparing radiocarbon ages of different organic fractions of the same sediment layer. Terrestrial macrofossils, such as leaf and wood fragments, are mostly deposited instantly as well as syn-sedimentary, and are therefore often used for estimating sedimentation ages.

In contrast, radiocarbon ages of bulk sedimentary organic carbon (OC) might reflect contributions from pre-aged SOC, which requires more time to reach the sediments from soils. The MTT of sedimentary organic fractions is determined by the age difference between the organic fraction and the deposition age (Douglas et al. 2018). The temporal evaluation of MTT of soil-derived OC fractions, such as leaf waxes, in sedimentary archives allows estimation of the influence of land-use change on the soil-carbon dynamics on the long-term but also investigating pre-anthropogenic conditions and effects of renaturation periods. This approach has, for instance, permitted the identification of periods of accelerated soil erosion (Smittenberg et al. 2006; Gierga et al. 2016; Douglas et al. 2018; Haas et al. 2019; 2020).

**Fig. 1:**
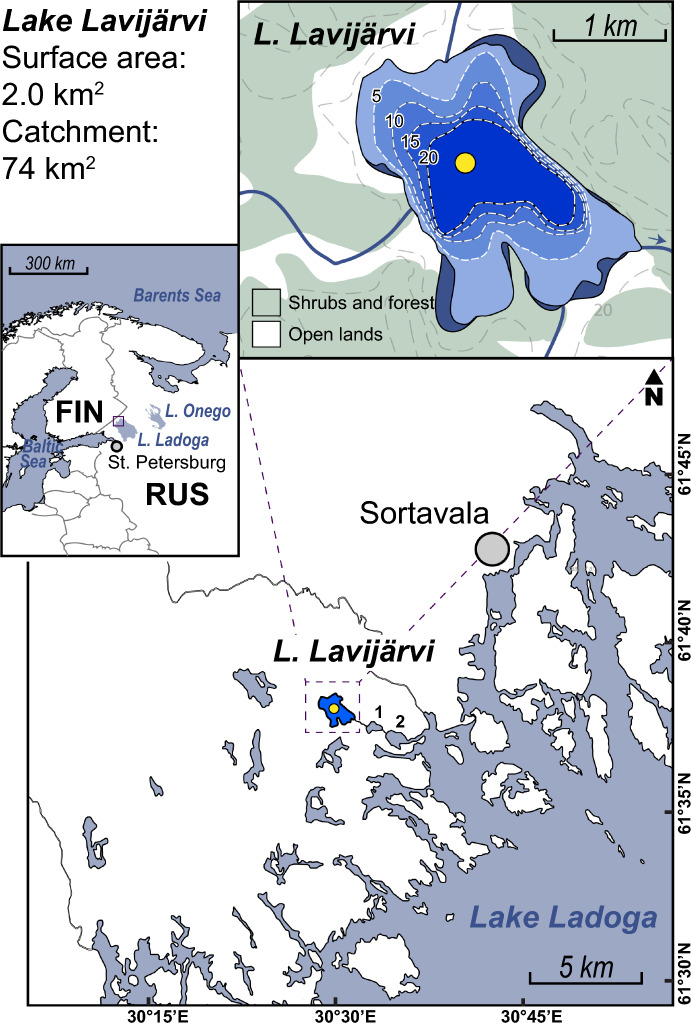
Location of Lake Lavijärvi in the Republic of Karelia, Russia. The coring location is shown as a yellow dot. The numbers 1 and 2 in the lower panel refer to Lake Polyakovo and the Otsoistenlahti Bay, respectively

In this study, we present a sedimentary record from Lake Lavijärvi (61°38.267’ N, 30°29.407’ E) a small 2.2 km^2^ lake located 5 km north of Lake Ladoga (Fig. 1), at an elevation of 5.9 m above sea level (m a.s.l.). It reaches a maximum depth of 26 m (Ludikova et al. 2024). The lake drains via a small stream to Lake Polyakovo (5.2 m a.s.l.) and finally to the Otsoistenlahti Bay of Lake Ladoga (~ 5 m a.s.l.). Kuznetsov et al. (2025) showed that in the early Holocene, Lake Lavijärvi was part of a larger basin, Lake Ladoga, but became isolated in the Mid-Holocene as a result of the termination of the Ladoga transgression, the formation of the Neva River, and the drop of Lake Ladoga to its present level after 3900 cal yrs BP.

Lake Lavijärvi is located within the Meyeri Thrust, a suture between Archaean crust of the Karelian Craton and Paleoproterozoic crust of the Svecofennian Orogen (Baltybaev et al. 1996). The transition between Archaean and Proterozoic crust occurs in the Raahe-Ladoga Zone. The Meyeri Thrust zone is mainly composed of garnet–biotite, garnet–(orthopyroxene)–cummingtonite, high-Al, cordierite paragneisses, and various granitoids (Baltybaev and Vivdich 2021). Abundant clayey soils are found in the catchment of Lake Lavijärvi (Simola 2000). There are no carbonate rocks in the catchment that could induce a hardwater effect.

The landscapes of the northern Lake Ladoga region are characterized by fjord-shaped lake shores, skerries, and bedrock ridges (Finnish *Selkä*—elongated, glacially shaped positive landforms). The study area includes a wide range of landform types, from high steep rock ridges to flat lacustrine plains and valleys. The land is covered by diverse forest vegetation—from southern taiga spruce forests and middle taiga spruce and pine forests (shrub-green moss and lichen-green moss types) to poor crowberry pine stands, close to northern taiga, on rocky lake shores. Large areas are occupied by agricultural lands on ameliorated soils developed on lacustrine and palustrine deposits, as well as by secondary forests on abandoned lands. This is one of the richest and most distinctive floristic regions of Karelia in terms of species composition (32 species) (Gnatvuk et al. 2011).

The cultural landscape of the study site originates from deforestation at the end of the Iron Age (Alenius et al. 2004). In the High Middle Ages, the northern Lake Ladoga area was disputed between Sweden and Russia. In 1323 CE, under the Orekhovo Peace Treaty, the lands were ceded to the Novgorod Republic (Russia). Between 1580–1597 CE and 1618–1721 CE, the lands were occupied by Sweden. From 1721 to 1917 CE, the territory was part of the Russian Empire. In 1917 CE, the government of the Russian Soviet Republic recognized the independence of Finland. After the victory of the USSR in World War II (WWII) in 1945, the Finnish history of the Ladoga region ended. Finland ceded the eastern part of Karelia to the Soviet Union and large parts of the population were evacuated. For more than 700 years, the ethnic composition of the Ladoga region changed repeatedly. The territory was inhabited and developed by Slavic and Finno-Ugric peoples. Ethnic and political development repeatedly led to changes in land-use practices.

The history of agriculture in the region spans from ancient slash-and-burn cultivation and cattle breeding to a developed farm economy in Finland, followed by large-scale Soviet collectivization and post-Soviet transformation. The most intensive agricultural period, accelerated by mechanization, occurred in the 1920s and 1930s (Miettinen et al. 2005). Intensive cereal and fodder cultivation was practiced during WWII until 1939 CE (Heikki et al. 1996; Simola 2000; Rautiainen et al. 2016). After WWII, agricultural activity was restored only in the 1950s (with migration from Russia, Belarus, Ukraine, and the Volga region), with a focus on dairy farming and livestock breeding. In the Soviet planned economy of the post-war period, fields formerly used for cereal cultivation were converted into pastures, and logging was banned (Rautiainen et al. 2016). Forests recovered and expanded continuously. With the fall of the Soviet Union in 1989 CE, the area of abandoned farmland increased, and the logging ban was lifted, though with only modest effect on forest composition (Rautiainen et al. 2016). Former meadows on clay lowlands became overgrown with forests (Isashenko 1996). The predominant succession pathway is: meadow—meadow with willow (*Salix* spp.) —deciduous forest—mixed spruce-deciduous forest. The European taiga generally promotes effective spontaneous reforestation. Currently, in the northwestern Ladoga region, deforestation, reforestation and regeneration occur alongside the restoration of forests on former Finnish arable land. There is a gradual shift toward spruce-dominated forests, eventually leading to more uniform landscapes (Isasenko 2018).

We applied common paleolimnological proxies in order to detect periods of accelerated soil erosion and evaluate their impact on the lake system and catchment soils but also to investigate the effect of relaxation of human pressure on the lake system. The bulk sediment geochemical and isotopic composition as well as XRF-derived elemental composition and MS served as the main scaffolding to capture environmental changes or land use (Haas et al. 2019). Radiocarbon (^14^C) dating of the bulk sediment fraction as well as on specific leaf-wax fractions (*n*-carboxylic acids) on the other hand allowed the analysis of changes in the transfer times of soil-derived OC (MTT_OC_ and MTT_LW_ for the bulk OC and the leaf waxes, respectively) in a historical to pre-historical context (Haas et al. 2020). In addition, the distribution of sedimentary leaf waxes, such as *n*-alkanes and *n*-carboxylic acids, was analyzed to identify potential sources of the sedimentary OM.

## Material and methods

### Sedimentary description

**Fig. 2:**
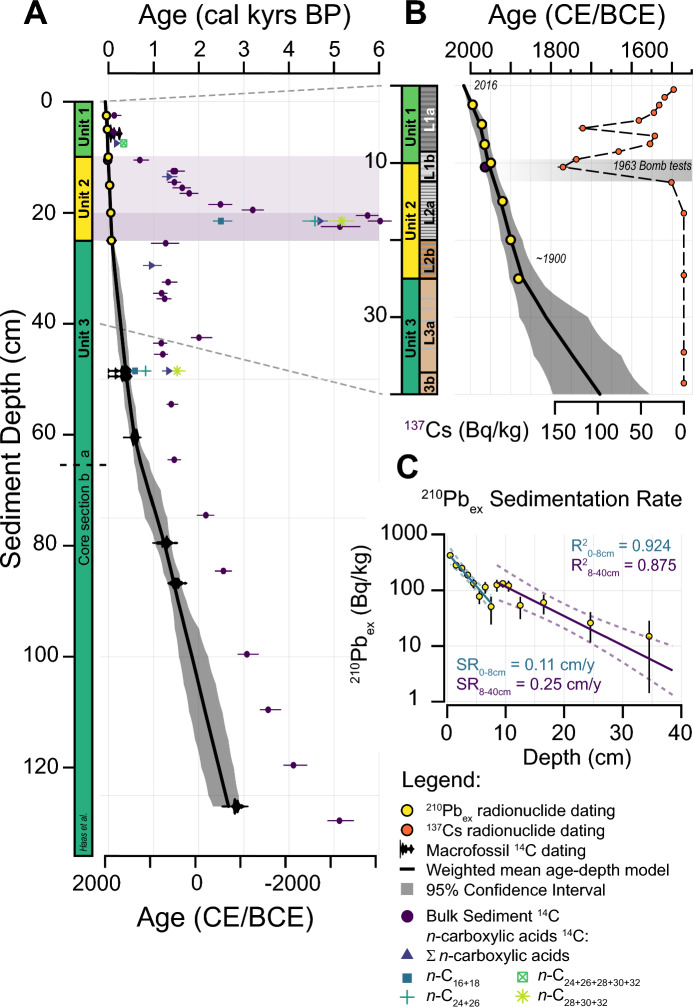
**A** Chronology of the Lake Lavijärvi sedimentary record (LAV16-05) based on macrofossil ^14^C and radionuclide dating, using Bayesian age-depth modeling (BACON by Blaauw and Christen (2011)). **B** Zoom into the age-depth model, showing lithological subsection on the y-axis. **C** Estimated sedimentation rate

A 135-cm long sediment core was retrieved from Lake Lavijärvi (LAV16-05) in March 2016, using a 63 mm diameter gravity corer, at a depth of 22 m (Fig. 1). The core was split open lengthwise in the Eawag Sedimentology laboratories before being visually described using macroscopic characteristics such as sediment colour, grain-size distribution and the presence of lamination and deformation structures (Fig. 2B and Appendix A). Grain-size measurements were performed at 2 cm resolution over the entire core using a Malvern laser-optical grain-size analyser. The sediment is mainly composed of fine silts. The mean grain-size distribution is composed of 5% sand (63 µm–2 mm), 80% silt (2–63 µm) and 15% clay (0.01–2.00 µm, *n* = 68 samples). The grain-size distribution is relatively stable through the entire core, except for higher clay content (reaching ca. 25%) in the lowermost part of the sediment core, at approximately 135–100 cm depth. Non-laminated and homogeneous sediments can be observed in the interval from 135 to 38 cm (Lithology 3b, L3b, Fig. 2B). Above 38 cm, a diffuse transition follows and the sediments are interrupted by alternating, fine laminations of a slightly darker and greenish color (regularly spaced, ca. every 2–5 mm, L3a). Starting from 24.5 cm a sharp transition towards red-brownish, detritus-rich laminations with alternating bright layers followed (L2b). At approximately 19.5 cm the brown laminations start to vanish and are replaced by finer and dark-greyish laminations above (L2a). Around 13 cm, the laminations become progressively thicker and more organic-rich (L1b). At 7–8 cm, there is a diffuse transition towards an interval with more bright and greyish laminae. From 8 cm to the core top, the sediments become very dark and organic rich and the laminations very prominent (L1a). Under the assumption of an annual lamination, we estimated for the first 8 cm a sedimentation rate of approximately 0.15–0.2 cm/yr (47 ± 7 counts). The lithostratigraphy of LAV16-05 compares well with the photograph of a Lake Lavijärvi sediment record published by Simola (2000).

### Chronology and unit assignment

The chronology of the 135-cm long sedimentary record of Lake Lavijärvi is based on eight age estimates derived from sixteen ^210^Pb and ^137^Cs radionuclide measurements and seven radiocarbon dates of macrofossil remains (twig, wood and leaf fragments) (Fig. 2A, Appendix B). Radionuclide measurements were carried out on a high-purity Germanium Well Detector (HPGe, Gamma spectrometer). For calculating sedimentation rates from the unsupported, i.e. excess, ^210^Pb fraction (^210^Pb_ex_) a constant rate of supply model was assumed following the method of Appleby (2008) (Fig. 2BC).

The radiocarbon measurements of Acid–Base-Acid treated macrofossils (after Hajdas et al. 1995) were performed on an Elemental Analyzer (EA) equipped Accelerator Mass Spectrometer (AMS), using the MIni CArbon DAting System (MICADAS) at the Laboratory of Ion Beam Physics of ETH Zürich (Ruff et al. 2010; Wacker et al. 2010). For evaluating the constant ^14^C contamination during sample processing (Welte et al. 2018), a brown coal blank of known ^14^C activity (Fm ~ 0) was monitored (a detailed summary of all macrofossil radiocarbon measurements is given in Appendix B). Bayesian age-depth modeling, including ages of both radiocarbon and radionuclide dating, was carried out using the BACON R code developed by Blaauw and Christen (2011) (Fig. 2). Mean sedimentation rates of 270 subsections (5 mm resolution) were calculated accordingly. The sedimentation rate of approximately 0.11 cm/yr estimated by ^210^Pb_ex_ (0–8 cm) is slightly lower compared to the sedimentation rate of 0.15–0.2 cm/yr estimated by varve counting. We thus increased the age error of the radionuclide anchor points by five years in order to account for this uncertainty (Appendix C).

The sediment core was subdivided into three units according to the historical background and the sedimentological description. Unit 3, from 1680 to 1870 CE, represents the pre-industrial era with homogeneous sediments (24.5–40 cm, L3a and b), Unit 2 the industrial period (12–24.5 cm, 1870–1950 CE) with detritus-rich and laminated sediments (L2a and b), and finally Unit 1 the post-WWII period (1950 CE—today) with organic-rich sediments (L1 a and b) between 0–12 cm.

### Magnetic susceptibility and XRF elemental composition

**Fig. 3:**
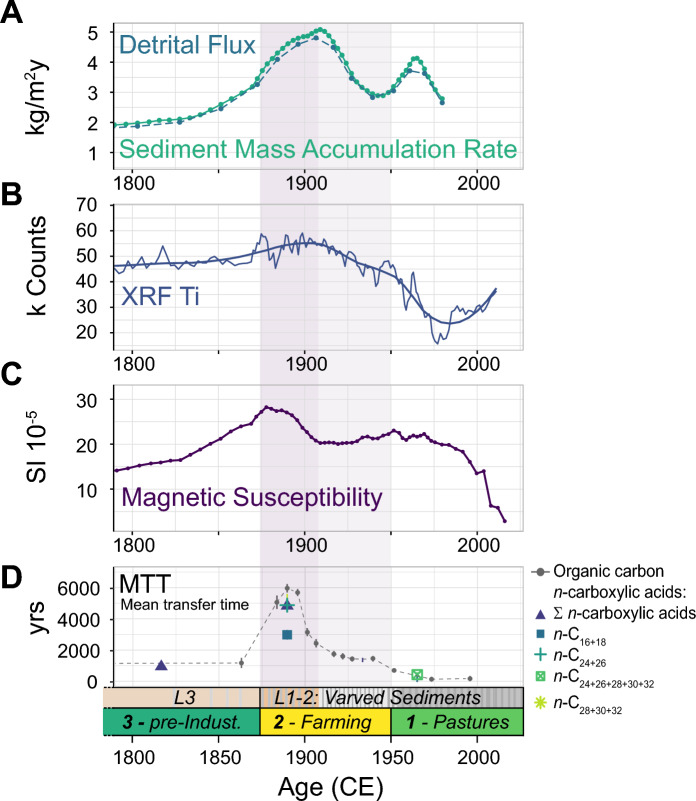
Paleolimnological proxies of soil erosion. **A** Sediment mass accumulation rate (MAR) and detrital flux. **B** Elemental abundance of titanium (XRF). **C** Magnetic susceptibility. **D** Mean transfer time of sedimentary organic carbon (MTT_OC_) and of leaf waxes (MTT_LW_). The legend on the bottom right indicates which chain lengths were pooled together for the compound specific radiocarbon dating

MS and gamma-density was measured on a Multisensor Core Logger (MSCL, Geotek) with a 5 mm resolution. XRF scans were performed on an Avaatech core scanner with 1 mm resolution. Sediment mass accumulation rates (MAR) and individual fluxes (Figs. 3, 2) were calculated using the sedimentation rates from the age-depth model, gamma density (MSCL), estimations about the dry bulk density, water content and porosity, following Müller et al. (2005).

### Bulk geochemical analysis

To track changes in the origin of the sedimentary OM we measured the TOC, total inorganic carbon (TIC), TN, total phosphorous (TP), and biogenic silica (bSi) content, but also the δ^13^C and δ^15^N isotopic signatures and the relative radiocarbon ages (^14^C) of the sedimentary OM. In total, 135 sediment samples (1 cm resolution) were freeze-dried and homogenized in a mortar before the geochemical measurements. Using a CNS Analyzer (EURO EA 3000) and titration coulometer (CM5015), TC, TN and TOC concentrations were measured. Sedimentary TP concentrations were quantified with a San +  + Flow Injection Analyzer from SKALAR using the method of Boltz and Mellon (1948). bSi concentrations (68 samples) were measured after Ohlendorf and Sturm (2008).

An EA-IRMS (EA Vario Pyro Cube by Elementar and IRMS by GV Instruments, Isoprime) was used to measure δ^15^N and δ^13^C values of 37 bulk sediment samples. A reference standard (Acetanilide #1) was used for quantification. Since TIC concentrations were zero throughout the sediment sequence, no acid pre-treatment was performed prior to the δ^13^C measurement. The δ^13^C signature of the bulk sediment thus represents the δ^13^C value of the OC. Repeated measurements of reference materials Acetanilide #1 (Schimmelmann, USA, δ^15^N =  + 1.18 ± 0.02) were used to determine the instrument precision, which was determined to be generally below 0.11 ‰ for δ^15^N. The offset between the measured and known δ^15^N values of the Acetanilide standard was on average 0.37. For δ^13^C, instrument precision during the runs was below 0.07 ‰ for Acetanilide #1, with an accuracy above 0.23. No corrections of the δ^15^N or δ^13^C values were performed.

Bulk sediment-radiocarbon dating was performed following an Acid–Base pre-treatment (Haas et al. 2019). A selection of 28 samples as well as processing blanks were weighted into combusted heat-treated Ag capsules. They were placed in a desiccator with HCl (37%), and NaOH (pellets) respectively, for 72 h at 60 °C. After folding the Ag caps containing the samples and blanks and placing them into new Sn boats, the measurement was carried out on the EA-AMS at ETH Zürich (MICADAS). NIST SRM 4990C (Oxalic acid II) was used as primary standard and phthalic anhydride as fossil blank. Additionally, we monitored and corrected the constant contamination introduced during the processing using Swiss standard soil (Fm = 1.06) and inhouse Swiss shale (Fm = 0) as processing blanks, according to Hanke et al. (2017). All bulk radiocarbon measurements are listed in Appendix B.

### Leaf-wax analysis

In the upper part of the core, leaf wax *n*-alkane and *n*-carboxylic acid distributions were examined at nearly 1 cm resolution (31 samples), covering the land-use period of interest (0–50 cm). We extracted the total lipid extracts (TLEs) from the ground and homogenized sediments (1 -5 g dry sediment) with 10 mL of 10% methanol in dichloromethane (DCM) using a microwave extraction system from Milestone (MLS 1200 mega, 2 min with 300 W at 70 °C and 5 min with 500 W at 70 °C). After extraction, the TLE was dissolved in *n-*hexane and loaded on an aminopropyl pipette column (LC-NH2 SPE Bulk, Sigma Aldrich, 1 g). To separate the target fractions, different solvents of increasing polarity were passed over the column. Fraction 1, containing the *n*-alkanes, was retrieved with 10 mL of *n*-hexane, fraction 2 with 4 mL of 20% DCM in *n*-hexane, fraction 3 with 4 mL of 10% acetone in DCM and fraction 4, containing the *n*-carboxylic acids, with 2% formic acid in DCM. The *n-*carboxylic acid fraction (fraction 4) was additionally methylated (derivatized) with 10 mL of 5% hydrochloric acid (32%) in methanol at 70 °C for 12 h. The organic phase (fatty acid methyl esters, FAMEs) of the reaction was eluted with two different solvents of increasing polarity over a silver nitrate-coated silica gel pipette column (Sigma Aldrich, 0.4 g) for further purification: fraction 1 with 2 mL of *n*-hexane and fraction 2 with 4.5 mL of 20% DCM in *n*-hexane. *n*-Alkane fractions and the derivates of the *n*-carboxylic acid fractions were identified by gas chromatography-mass spectrometry (GC–MS, 5977B MSD, Agilent Technologies). An external *n*-alkane (*n*-C_7-40_) and *n*-carboxylic acid standard (*n*-C_16+22+24+28_) was used for quantification of the individual homologues. The average chain lengths (ACL) were determined after Poynter and Eglinton (1990) and the carbon preference indices (CPI) after Fisher et al. (2003). Formula and a table summarizing the leaf-wax distribution are shown in Appendix D for the *n*-carboxylic acids and Appendix E for the *n*-alkanes.

### Compound-specific radiocarbon analysis

Six sediment samples of 3-cm thickness (ca. 10 g of dry and homogenized sediment) were selected to obtain sufficient *n*-carboxylic acids for compound-specific radiocarbon dating. The TLE was saponified (2 h at 70 °C) using 10 mL of 0.5 M potassium hydroxide in methanol. Subsequently, 5 mL of water was added to the mixture. The neutral fraction (F1) was retrieved by repeatedly adding 10 mL of *n*-hexane and performing a liquid–liquid extraction (four times). The residue was acidified to a pH of 2 by adding concentrated hydrochloric acid (37%). The acid fraction (F2) was retrieved by repeatedly adding 10 mL of 20% DCM in *n*-hexane and performing a liquid–liquid extraction (four times). F2 was additionally methylated (derivatized) using 10 mL of 5% hydrochloric acid (32%) in methanol of known radiocarbon activity at 70 °C for 12 h. A liquid–liquid extraction with four times 10 mL of *n*-hexane was performed to retrieve the organic phase of the reaction (Fatty acid methyl esters, FAME). For further purification, the FAME fraction was eluted over a silica gel pipette column (1% water deactivated silica gel) using different solvents of increasing polarity: the first fraction containing the target compounds (FAMES) was retrieved with 4 mL of 30% *n*-hexane in DCM. The second fraction (Flush) was retrieved using 4 mL of DCM. A quantification on the GC-FID showed that no further purification was necessary to continue with the isolation of individual components.

Specific *n*-carboxylic acid homologues *n-*C_16_, *n-*C_18_, *n-*C_24_, *n-*C_26_, *n-*C_28_, *n-*C_30_ and *n-*C_32_ of three samples were isolated using a GC-FID (7890A, Agilent) equipped with a preparative fraction collector (PFC1, Gerstel). The individual compound fractions were recovered from the PFC glass traps with 1 mL DCM and tested on a GC-FID for its mass and purity. The compound fractions were transferred with 100 µL of 10% *n*-hexane in DCM into EA tin capsules (Elementar, 0.025 mL). Several individual compound fractions were combined in order to obtain enough quantity for EA-AMS radiocarbon dating (> 20 µg C). Additionally, the bulk *n*-carboxylic acid fraction (Σ *n*-carboxylic acids, containing every chain length) of these three samples was analyzed, along with two additional samples.

Radiocarbon measurements of five bulk *n*-carboxylic acid fractions and eight compound-specific fractions were performed on an EA accelerated mass spectrometer (EA-AMS) at the Laboratory for Ion Beam Physics of ETH Zürich, Switzerland (Ruff et al. 2010; Haghipour et al. 2018).

To monitor recoveries and ^14^C backgrounds, specific standards with known ^14^C concentration were processed. Samples that went through compound separation (GC-PFC) were corrected for the constant background contamination with GC-PFC processed standards. Bulk *n*-carboxylic acid fractions were corrected with unprocessed standards. Constant contamination was corrected following Hanke et al. (2017). All radiocarbon values are corrected for procedural blanks with error propagations. Procedural blanks yielded 0.80 ± 0.10 μg C with an Fm value of 0.67 ± 0.15 for GC-PFC injections. The carbon isotopic values of n-FAMEs were also corrected for the derivative carbon from MeOH. The obtained radiocarbon ages were calibrated with IntCal 13 (Reimer et al. 2013). A table summarizing the data of the CSRA is given in Appendix F.

## Results

### Soil-erosion indicators

During the pre-industrial period (Unit 3, until 1870 CE) soil-erosion indicators such as the total sediment MAR, flux of detrital material, XRF titanium abundance (Ti) and MS were at a relatively low level (Fig. 3).

Towards the transition to Unit 2 (1870, beginning of the industrial period), the soil-erosion indicators began to rise and reached a maximum in the early part of Unit 2 (ca. 1900 CE). The sediment MAR peaked with 5.1 kg/m^2^/yr around 1910 CE, mostly containing detrital (i.e., inorganic) material from the surrounding catchment (4.8 kg/m^2^/yr, 94% of MAR). The MS and Ti values reached their maxima during the same period but slightly earlier around 1880 CE and 1900 CE, respectively (Fig. 3).

The relative ages of the sedimentary OM compared to the age-depth model, i.e. MTT_OC_ (Appendix G), show a correlation with the excursions of MAR, MS and Ti counts (1880–1910 CE) and revealed enhanced input of old OC. Starting around 1880 CE, MTT_OC_ started to rise from values of approximately 1080 years (MTT_OC_ mean of Unit 3, *n* = 4) up to a maximum of 5960 years in 1890 CE Thus, within approximately only 30 years, the age of the sedimentary OC increased by 4880 years compared to the deposition age. After this steep ascent, MTT_OC_ sank continuously, almost exponentially, during the rest of Unit 2. Similar decreasing trends could also be observed in the MS and MAR signal. Towards the end of Unit 2 (1950 CE), most soil-erosion indicators reached similar levels to those of Unit 3, the pre-industrial period.

The MTT_LW_ (Appendix F) matches well the evolution of MTT_OC_, in particular the bulk fraction of the leaf waxes. During the peak in old soil input around 1890 CE, long-chain leaf waxes reveal MTT of 4879 ± 261 years (C_24_ + C_26_) and 5422 ± 188 years (C_28_ + C_30_ + C_32_), whereas the shorter chain length (C_16_ + C_18_) only reach a MTT of 2982 ± 305 years (Fig. 3D). The bulk leaf-wax fraction has a MTT of 4831 ± 245 years.

With the onset of Unit 1 (1950—today), MAR started to increase again and reached a temporal maximum of 4.1 kg/m^2^/yr by 1962 CE. In contrast, MS, Ti and MTT_OC_ showed decreasing trends, reaching even lower values than those of Unit 3 and 2.

### Bulk geochemical composition

**Fig. 4:**
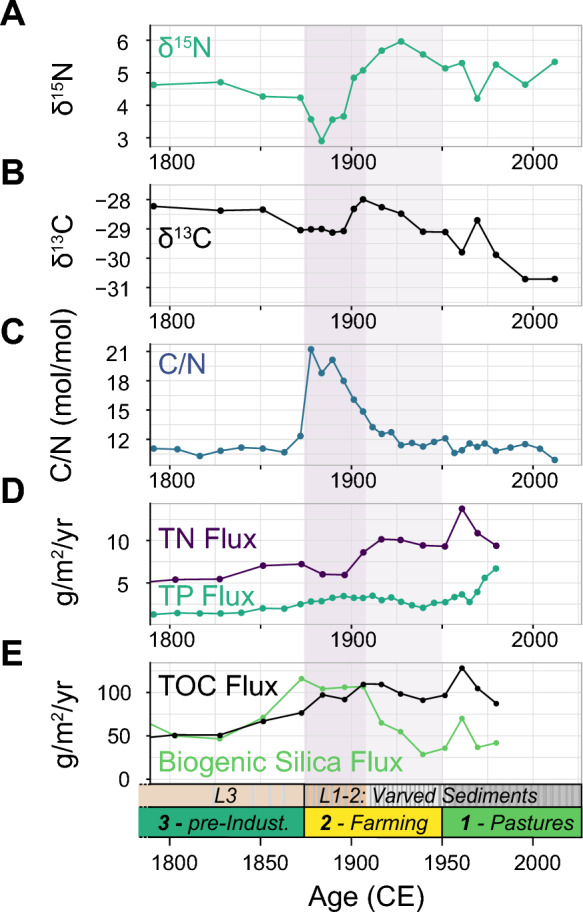
Source assessment of sedimentary organic matter. **A** and **B** show the δ^15^N and δ^13^C values (‰) of the sedimentary organic matter. **C** indicates atomic C/N ratio, **D** the TN and TP flux and **E** the TOC and biogenic silica flux (g/m^2^/yr)

The pre-industrial era (Unit 3) was characterized by relatively low and stable fluxes of TOC, bSi, TN and TP (Fig. 4). The C/N ratio was constantly low as well at approximately 11.1 (1680–1870 CE, *n* = 15). The stable isotopic (δ^15^N and δ^13^C) composition of the sedimentary OM showed a slight decreasing trend towards the transition to Unit 2 (1870 CE), with average values at 4.7 ‰ and -28.2 ‰, respectively (*n* = 5).

The bulk geochemical composition of the sediments of Unit 2, the industrial period (1870–1950), differed significantly from those of the previous Unit 3. TOC, bSi and TP flux showed elevated values from 1870 to 1950 CE TN flux, on the other hand, showed a temporal minimum of 6.0 g/m^2^/yr at 1890 CE, together with a sharp peak in the C/N ratio (C/N of 21.2 at 1890 CE). Within the same period, δ^15^N and δ^13^C values started to decrease to 2.9 ‰ and -29.1 ‰, respectively. Towards the end of Unit 2, TOC and TN inputs were increasing, whereas bSi and TP flux were diminishing again. The C/N ratio was decreasing, reaching similar values to those during Unit 3. During the same period δ^15^N and δ^13^C were increasing simultaneously and reaching a temporal maximum of 6.0 ‰ and -28.0 ‰ (approximately 1930 and 1910 CE, respectively).

Unit 1, the post-WWII period, was characterized by relatively high TOC, TN and TP fluxes and the C/N ratio (mean, 11.0) approaches pre-industrial values (*n* = 10). δ^15^N values of the sedimentary OM remained relatively high at approximately 4.9 ‰ (n = 5) during Unit 1, whereas δ^13^C decreased continuously to -30.7 ‰ by 2012 CE.

### Sedimentary leaf-wax distribution

**Fig. 5:**
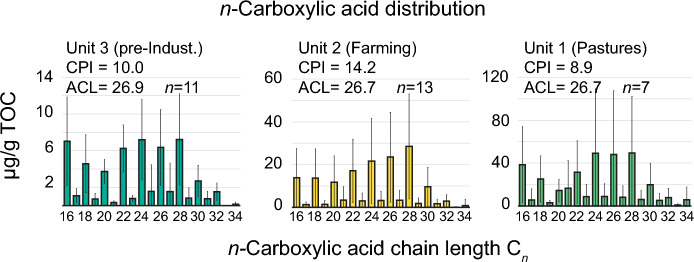
Boxplots of n-carboxylic acid homologue distribution grouped by Unit 1–3. *Note* that each plot is on a different y-axis

During Unit 3 (pre-industrial period, 1680–1870 CE) the mean *n*-carboxylic acid homologue pattern showed a bimodal distribution with maxima on *n*-C_16_ and *n*-C_24_. (Fig. 5). The mean distribution in Unit 2 (industrial period, 1870–1950 CE) differed significantly from the previous unit. The bimodal structure of the *n*-carboxylic acid distribution was shifted towards longer chain lengths and the concentrations relative to TOC increased by approximately a factor of four. *n*-C_28_ carboxylic acid represented the most abundant homologue during Unit 2. The mean *n*-carboxylic acid distribution of Unit 1 (Post-WWII period, 1950 CE—today) had a similar bimodal appearance to that of Unit 3 (pre-industrial period, Fig. 5), with peaks around the *n*-C_16_ and the *n*-C_24-28_ homologues. Interestingly in Unit 1, the *n*-C_16_ homologue was clearly the most abundant, but also the *n*-C_17_ and *n*-C_18_ were relatively more abundant than in Unit 3. The *n*-alkanes homologue pattern remains similar throughout the core, with *n*-C27 being the most abundant homologue.

**Fig. 6:**
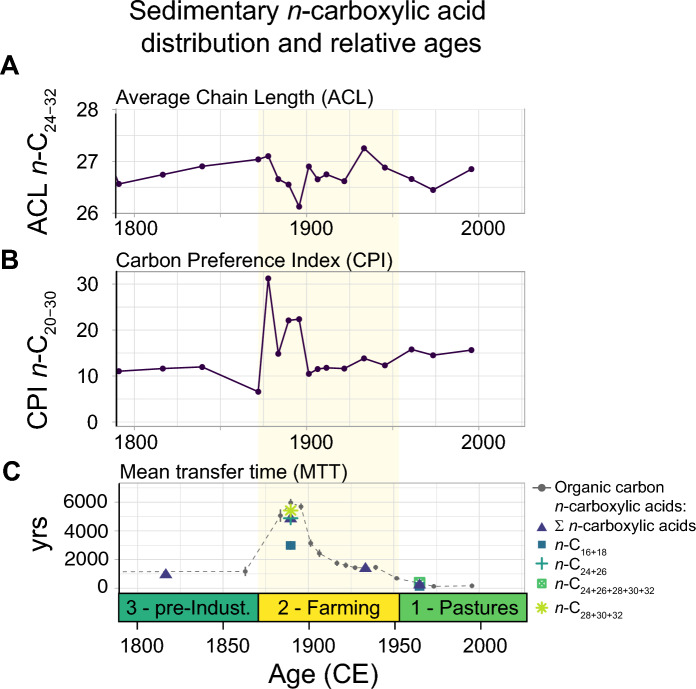
ACL (**A**), CPI (**B**) of the n-carboxylic acid, and MTT of the bulk OC and leaf waxes (**C**)

The temporal evolution of the ACL and CPI of the *n*-carboxylic acids revealed relatively stable values during Unit 3 (Fig. 6). At the beginning of Unit 2, however, ACL decreased substantially from 27.1 to a temporal minimum of 26.1 (1895 CE) in approximately 20 years. During the same period, CPI increased and showed a peak of 31.2 at 1880 CE (Fig. 5). The excursions of ACL and CPI during Unit 2 compare well to those of the soil-erosion indicators (Fig. 3) and the bulk geochemical composition, e.g. the C/N ratio (Fig. 4). During the second half of Unit 2 and Unit 1, CPI values decreased again. ACL, on the other hand, showed a slight increasing trend during the second half of Unit 2 (until ca. 1930 CE) and a decreasing trend during Unit 1. The temporal evolution of the *n*-alkane ACL, on the other hand, reveals an increase towards the core top starting in Unit 2. The *n*-alkane CPI remains relatively stable throughout, except for a minimum in the middle of Unit 3, and higher values in the core top.

## Discussion

### Initial land-clearing pulse

At the beginning of the last century, industrialization and an intensive farming period started in Southern Karelia. Arable field cultivation replaced slash and burn cultivation, the previously dominant land-use activity in Karelia (Simola 2000; Alenius et al. 2004). During the first half of the twentieth century, until ca. 1939 CE, intensive cereal and fodder cultivation was practiced in Karelia. During this time period, substantial deforestation took place to clear space for arable fields (Rautiainen et al. 2016).

A deforestation signal could explain the sharp increase in the C/N ratio (maximum of 21.2, 1890 CE, Fig. 4), since fresh terrestrial plant material typically has a high C/N ratio (Enters et al. 2006). The δ^13^C and δ^15^N values of the sedimentary OC reacted in the same time interval with a drop to lower, more depleted values (-29.1 ‰ for δ^13^C and 2.9 ‰ for δ^15^N, ca. 1900 CE, Fig. 4), characteristic for terrestrial OC (Stuiver 1975; Talbot 2002).

The sedimentary *n*-carboxylic acid distribution provides further indications for a predominantly terrestrial source of the sedimentary OM. The lipids deposited at the very beginning of Unit 2 were characterized by relatively high CPI (maximum of 31.2, 1880 CE), indicating that the leaf waxes predominantly originated from a higher plant source and were not subject to notable microbial degradation or petrogenic inputs (Bush and McInerney 2013; Tao et al. 2015). The absence of an unresolved complex mixture (UCM) in the *n*-alkane fractions and the relatively high CPI exclude a potential influence of fossil fuel-derived lipids.

The increase in C/N ratio at the very beginning of Unit 2 as well as the high CPI value precede the increase in MTTs and would be consistent with a pulse of fresher, younger terrestrial plant-derived OM from land clearing/deforestation at the beginning of the period of intensive agriculture.

### Intensification of agriculture

The land-use change and intensification of agriculture is clearly reflected in simultaneous increases of MAR, MS and XRF Ti (Fig. 3) since the beginning of Unit 2, peaking by ca. 1900 CE, due to enhanced input of detrital soil material (Haas et al. 2019). The timing of this sedimentary unit was set several years later in the 1920s–1930s according to the age model of Miettinen et al. (2005), based on varve counting. The 95% confidence interval of the mean age of 1900 CE is relatively wide and ranges from 1875 to 1925 CE according to our age model. By estimating and including an age error of ca. 10 years (e.g., from varve counting, coring etc.) in the model of Miettinen et al. (2005), the events match within uncertainty.

During the same period, a prominent radiocarbon anomaly in the bulk sediment and the leaf-wax ages was detected (1890 CE, MTT_OC_ of 5960 years, MTT_LW_ up to 5422 years, Figs. 2, 3). At its peak, the sedimentary OC was approximately 5000 years older than during Unit 3 (representing baseline conditions of ca. 1000 years MTT_OC_, 1680–1870 CE, Figs. 2, 3). We assume that soil-erosion processes were mainly responsible for the mobilization and deposition of old OC, including leaf waxes stored in the soil, in Lake Lavijärvi (Edwards and Whittington 2001; Haas et al. 2019; 2020). While the radiocarbon measurements performed on sedimentary TOC represent a mean of the ages of various different OC fractions from different allochthonous and autochthonous sources, the very similar ages detected in the leaf-wax fractions, in particular the long chain homologues (ε *n*-C_24_), support the hypothesis that the ageing of the sedimentary OC originated from a pre-aged allochthonous OC source (Gierga et al. 2016; Haas et al. 2020).

During Unit 2, a drop in ACL was visible (26.1, 1895 CE). Aquatic plants like microalgae or macrophytes predominantly produce middle and short-chained homologues (Ficken et al. 2000; Sachse et al. 2004), which might be responsible for the minima in ACL. In the case of Lake Lavijärvi, the slight decrease in ACL would correlate well with the beginning of varve formation and eutrophication (Miettinen et al. 2005). On the other hand, we also observed a higher input of long-chain *n*-C_28_ acids during Unit 2 (Fig. 5), characteristic for enhanced terrestrial leaf-wax input. We, therefore, suspect a mixed *n*-carboxylic acid signal consisting of inputs from both aquatic and terrestrial sources during Unit 2.

### Rising eutrophication despite stabilization of catchment soils

Towards the second half of Unit 2, ca. 1920–1950 CE, markers of high soil erosion (MAR, MS, XRF Ti, MTT_OC_) were still evident, but were slowly starting to decline. The reduction in soil erosion was particularly evident in MTT_OC_. Within an interval of approximately 50 years, MTT_OC_ and MTT_LW_ decreased to nearly background level (1420 years and 1364 years, respectively), indicating a continuous change in the sedimentary OM source. We believe stabilization of catchment soils is the main driver for decreasing MTT.

However, enhanced input of a young OC source, e.g. from aquatic primary production, possibly contributed to the signal. With the wash out of soil erosion products and the entry of substantial amounts of nutrients, primary production and eutrophication increased during the first half of the twentieth century. The effect of growing eutrophication was also evident in the diatom assemblages (Miettinen et al. 2005), which indicated up to five times higher phosphorous concentrations in the lake water (TP of 45 µg/L) during the period of intensive land use.

We could observe similar indications. TN, TP as well as TOC flux showed an increasing trend towards the present. The C/N ratio declined with a similar gradient as MTT_,_ suggesting a higher input of nitrogen compared to OC. OM in mineral soils generally has relatively low C/N ratios due to the presence of OM derived from microbial processing (Enters et al. 2006). During the same time interval, δ^13^C values slightly increased, while the δ^15^N value of the OM increased to 6 ‰ at 1930 CE, after the temporal minimum of 2.9 ‰ during the deforestation pulse (approximately 1890 CE). This ~ 3 ‰ increase suggests a major change in the N cycle. In lake sediments, nitrogen is mainly derived from aquatic sources (Meyers and Teranes 2002). We assume that higher productivity with higher nutrient utilization led to these higher δ^15^N values. Alternatively, denitrification processes might have occurred in the water column due to ongoing primary production and anoxia, leading to an ^15^N-enriched residual dissolved inorganic nitrogen (DIN) pool.

### The post-war period

After WWII, the region north of Lake Ladoga (Southern Karelia) was ceded to the Soviet Union, which led to a population collapse in the region after most of the Finnish population left their villages and abandoned their lands (Rautiainen et al. 2016). Rautiainen et al. (2016) estimated that agricultural land decreased by half from 18 to 9% with the introduction of the Soviet planned economy. In Southern Karelia, the nature of farming changed from grain cultivation to cattle raising.

In the sediment record of Lake Lavijärvi, this post-war period is preserved in the sediments of Unit 1 (1950 CE—today). The cessation of field cultivation is reflected in the further decline of soil erosion (MS, XRF-Ti, MTT_OC_, MTT_LW_) and the continuation of the trends of the organic fluxes, such as increase in TOC, TN, TP or the C/N ratio. MTT_OC_ decreases to below background levels during Unit 1 (1950 CE—today), which could be attributed to low soil OC input due to soil-stabilization processes. Rautiainen et al. (2016) described that forests started to recover after the cessation of agriculture and expanded onto previous farmlands due to a logging ban by the Soviet Union. We assume that forest recovery was an important driver for soil-stabilization processes in the catchment, consequently reducing the export of soil OM (decreasing MTT_OC_ trend). However, primary production could also have had a similar effect on MTT_OC_ by diluting the sedimentary OC with relatively young (in-situ produced) OC (Haas et al. 2019). This assumption would be supported by the sedimentary lipid distribution and isotopic composition. The mean *n*-carboxylic acid distribution of Unit 1 was similar to that of Unit 3 (pre-industrial) and showed a higher contribution of middle to short-chained homologues (Fig. 5). This lipid distribution would, therefore, suggest a higher input of aquatic OM (relative to Unit 2), which is also supported by higher δ^13^C and δ^15^N values. However, these parameters showed a slight decreasing trend at the onset of Unit 1 (1950 CE), potentially indicating a recovery towards mesotrophic conditions. The bSi fluxes also showed relatively low values around 1950 CE, in comparison to Unit 2. We therefore assume that the decrease in MTT_OC_ mostly reflects soil stabilization. Alternatively, the incorporation of bomb ^14^C into plant biomass would also lower the MTT_OC_. Bomb ^14^C refers to the significant temporary increase in atmospheric ^14^C caused by above-ground nuclear weapons testing which started in the 1950s.

Miettinen et al. (2005) also described signs of recovery from eutrophic conditions, correlated with the observed decrease in δ^15^N values. They observed a change in the diatom assemblage from eutrophic to more oligo-mesotrophic diatom species and reported that the associated diatom-inferred phosphorous concentrations in the lake water declined continuously until today. Moreover, we observed increased sedimentary TP deposition during the same time period (1950 CE—today), suggesting that large fractions of the phosphorous load in the water column were sequestered in the sediments. The relatively high sedimentation rates as well as the shallow water depth might have favored the burial of excess nutrients (Miettinen et al. 2005). However, the lithology of Unit 1 is still characterized by varved sediments, implying that anoxic conditions prevailed at the coring location since the period of industrialization until today. This leads to the presumption that nutrients like phosphorous and nitrogen might still be accessible for primary producers in Lake Lavijärvi due to an internal release from the lake sediments. Under anoxic conditions OM is decomposed continuously, leading to release of DIN and dissolved reactive phosphorous (DRP) which diffuse into the water column (Burger et al. 2007).

## Conclusion

The land-use history of Southern Karelia during the past 300 years is well preserved in the sedimentary record of Lake Lavijärvi. Lake Lavijärvi shares the fate of many lakes around the world that are struggling with eutrophication and hypoxia due to past land-use legacies. In the case of Lake Lavijärvi, however, the initial intensive agricultural phase that occurred in the nineteenth century was followed by a period characterized by a relaxation of human pressure, giving the opportunity to study the impact of renaturation processes of catchment soils on lake ecosystems.

With the start of industrialization and the mechanization of agriculture, we noticed an initial pulse of fresh terrestrial plant-derived OM as observed in the high C/N ratio and high CPI, followed by increasing indications for soil erosion, evident in peaking MS, XRF-Ti, MAR, MTT_OC_ and MTT_LW_ signals. We assume that land clearing and land-use change had a substantial impact on the catchment soils during this period, indicated by an acceleration of soil-carbon-residence times (MTT) through the wash out of pre-aged SOC and leaf waxes. With the soil-erosion products, also nutrients, such as phosphorous and nitrogen, were washed into the lake, resulting in varve formation due to eutrophication and hypoxia from which the lake has yet to fully recover.

After WWII, Finland ceded the region to the Soviet Union and large parts were depopulated. Soil erosion and the export of SOC declined in ca. 30 years to background levels due to the relaxation of human pressure (land abandonment) and forest regrowth. While soil erosion had stabilized in the catchment, the lake system experienced long-term effects of the past land-use legacy. Even though the trophic level of Lake Lavijärvi improved during the post-war period (lower bSi flux, δ^15^N and δ^13^C), anoxic conditions at the lake floor persist, evidenced by varve formation. We assume that an internal release of nutrients from the lake sediments to the water column slows the recovery to baseline conditions before 1870 CE.

The lake history of Lavijärvi suggests that reduction of nutrient sources, particularly sourced from soil erosion, is essential for lake mitigation from eutrophication. We could also demonstrate that land-use legacies exert a major influence on freshwater ecosystems decades later.

## Supplementary Information

Below is the link to the electronic supplementary material.

Supplementary file1 (JPG 461 kb)

Supplementary file2 (XLSX 344 kb)

## Data Availability

The data generated in this study is available in the Supplementary material.
