# Supplementary figures and images for: An example of land-use relaxation and its effect on soil stabilization in the catchment of Lake Lavijärvi, Russian Karelia: insights from multi-proxy sediment analysis
Source: J Paleolimnol. 2026 Jun 5;74(3):14. doi: 10.1007/s10933-026-00394-2 (PMC13241461; doi:10.1007/s10933-026-00394-2)

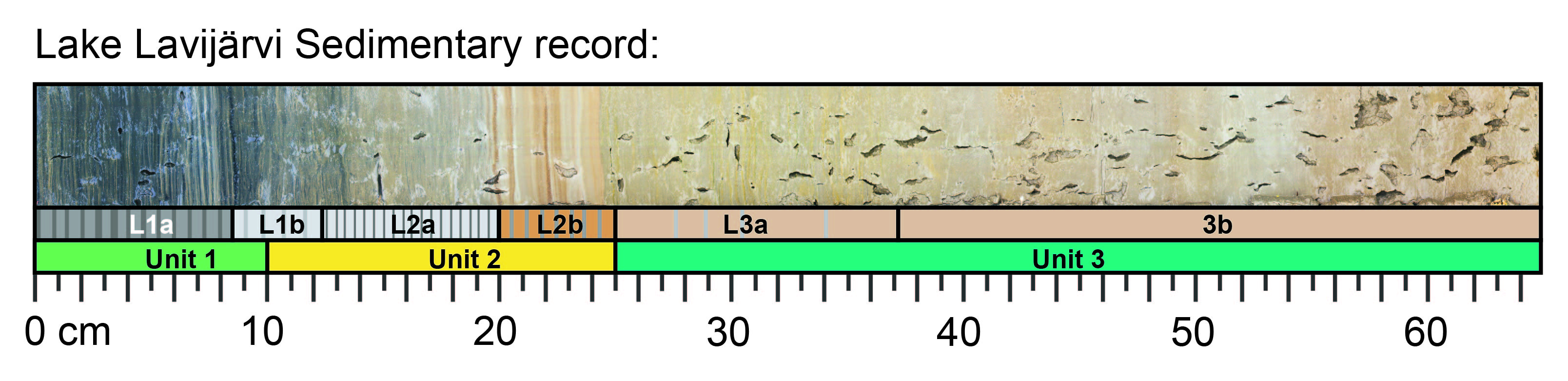

Supplement: Supplementary file 1 — Supplementary file1 (JPG 461 kb) [file 10933_2026_394_MOESM1_ESM.jpg]
